# Cumulative Extreme Events Threaten Penguin Habitats Across the Southern Hemisphere

**DOI:** 10.1111/gcb.70562

**Published:** 2025-10-23

**Authors:** Míriam Gimeno, Francisco Ramírez, Marta Coll, Andre Chiaradia, Camila Artana

**Affiliations:** ^1^ Institut de Ciencies del Mar Recursos Marins Renovables Barcelona Spain; ^2^ Departament de Biologia Evolutiva, Ecologia i Ciències Ambientals, Facultat de Biologia Universitat de Barcelona (UB) Barcelona Spain; ^3^ Ecopath International Initiative (EII) Barcelona Spain; ^4^ Conservation Department, Phillip Island Nature Parks Cowes Victoria Australia; ^5^ School of Biological Sciences Monash University Clayton Australia; ^6^ Laboratoire LOCEAN‐IPSL Sorbonne Université (UPMC, Université Paris 6), CNRS, IRD, MNHN Paris France

**Keywords:** air‐breathing marine fauna, climate change, ecosystem sentinel, extreme events, extreme precipitation, extreme winds, heatwaves, marine heatwaves, seabirds, Southern Hemisphere

## Abstract

Upon the rise in the intensity, duration and frequency of extreme events threatening life on Earth across land and ocean, there is a need to select ecologically significant areas for targeted management interventions. This study assesses the spatial overlap of multiple extreme events and calculates the cumulative magnitude faced across the Southern Hemisphere during the last decades, focusing on the 18 species of penguins as ecosystem sentinels that rely on terrestrial and marine environments. Our analysis identifies African, Snares, Emperor, Adélie and Galápagos as the penguin species experiencing the highest cumulative values of extreme events. Additionally, when looking at the trends, we identify that all penguin species, except the Galápagos penguin, will face a potential increase in extreme events if current trends are maintained. This study carries a crucial message in conservation, establishing a spatial framework that allows sounding the alert in ecologically important areas to reduce vulnerability during present and future extreme events.

## Graphical abstract

This study examines how multiple extreme events overlap across the Southern Hemisphere, focusing on hotspots of 18 penguin species across both land and sea. African, Snares, Emperor, Adélie and Galápagos penguins experience the greatest combined exposure, and most species are projected to face more frequent extremes if current trends persist. The results highlight priority areas for conservation to help protect penguin populations and the ecosystems they represent.

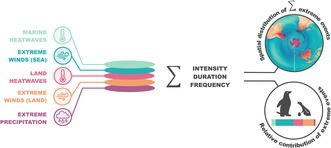

## RESUM

A causa de l'augment de la intensitat, la durada i la freqüència d'esdeveniments extrems que amenacen la vida al planeta tant a terra com a mar, hi ha lanecessitat de seleccionar àrees ecològicament rellevants per tal de focalitzar en aquestes àrees les mesures de gestió. Aquest estudi avalua la superposició espacialde múltiples esdeveniments extrems i calcula la magnitud acumulada d'aquests esdeveniments a l'hemisferi sud durant les últimes dècades, centrant‐se en elspingüins com a sentinelles de l'ecosistema, ja que, aquests es troben tant en ecosistemes terrestres com marins. Per tant, la nostra anàlisi destaca el pingüí africà,de Snares, l'Emperador, l'Adèlia i el de les Galápagos com les espècies que experimenta els valors acumulatius més alts d'esdeveniments extrems. A més, a l'horade mirar les tendències, identifiquem que totes les espècies de pingüins, excepte el pingüí de les Galápagos, suggereixen un augment potencial d'esdevenimentsextrems si es mantenen les tendències actuals. Aquest estudi porta un missatge crucial en la conservació, establint un marc espacial que permet detectar zonesecològicament importants que es troben afectades i, per tant, zones on cal focalitzar esforços per tal de reduir la vulnerabilitat durant els esdeveniments extremspresents i futurs.

## 1 Introduction

Extreme events are weather events characterised by values that deviate significantly from the mean climatology and persist for a certain period at specific locations and times of the year (IPCC 2021). These events, such as unusually high temperatures, heavy precipitation or floods, are unevenly distributed across the globe (Lee et al. 2023). From an ecological perspective, extreme weather events can act as disturbances, potentially altering the functionality and stability of ecosystems (De Luzinais et al. 2024; McPhillips et al. 2018). Although the precise impact of these disturbances remains debated (Rovira et al. 2024), an increasing body of research highlights their significant socio‐ecological consequences, which span from global to regional scales (Artana, Capitani, et al. 2024; Lee et al. 2023; Newman and Noy 2023; Smith et al. 2021; Turner et al. 2020). For example, intense storms or extreme heatwaves, even for a short period of time, can cause abrupt species mortality events (Holt and Boersma 2022; Wolfaardt et al. 2012). Prolonged extreme events in sea surface temperatures or winds at sea are commonly linked to changes in prey distribution, which may cause adults to arrive in worse conditions for the breeding season or lead to the abandonment of the offspring, gradually disrupting birth‐to‐mortality ratios (Piatt et al. 2020; Rebstock and Boersma 2018; Saraux et al. 2016). With the increasing frequency of extreme events (Oliver et al. 2018), such disturbances could have long‐lasting effects, such as reduced recruitment and slower growth rates (González‐Trujillo et al. 2023). This is particularly concerning for species with small populations or restricted distributions, as their ability to recover from disruptions is limited (Frederiksen et al. 2008; Sepúlveda et al. 2020).

Numerous recent studies have examined individual extreme events on large scales (Boers et al. 2019; Sen Gupta et al. 2020) and their impacts on various species and ecosystems (Cohen et al. 2020; Harris et al. 2018). However, the interactions among these events have received considerably less attention. Such interactions may act synergistically, amplifying their effects on ecosystems and species (Turner et al. 2020). For instance, rising temperatures linked to heatwaves can increase atmospheric moisture retention, thereby enhancing the likelihood of extreme precipitation events (Trenberth et al. 2003; You and Wang 2021). At sea, extreme temperature events are often accompanied by extremes in ocean acidity, oxygen depletion and low chlorophyll concentration (e.g., Gruber et al. 2021; Le Grix et al. 2021). Moreover, extremely low fish biomass has been shown to result from the combined effects of marine heatwaves and exceptionally low chlorophyll levels, rather than from one of those events alone (Le Grix et al. 2023). Despite growing concerns over these interactions, no previous research has systematically integrated assessments across multiple extreme events occurring both on land and at sea to identify ecologically significant areas most at risk from their combined effects.

**FIGURE 1:**
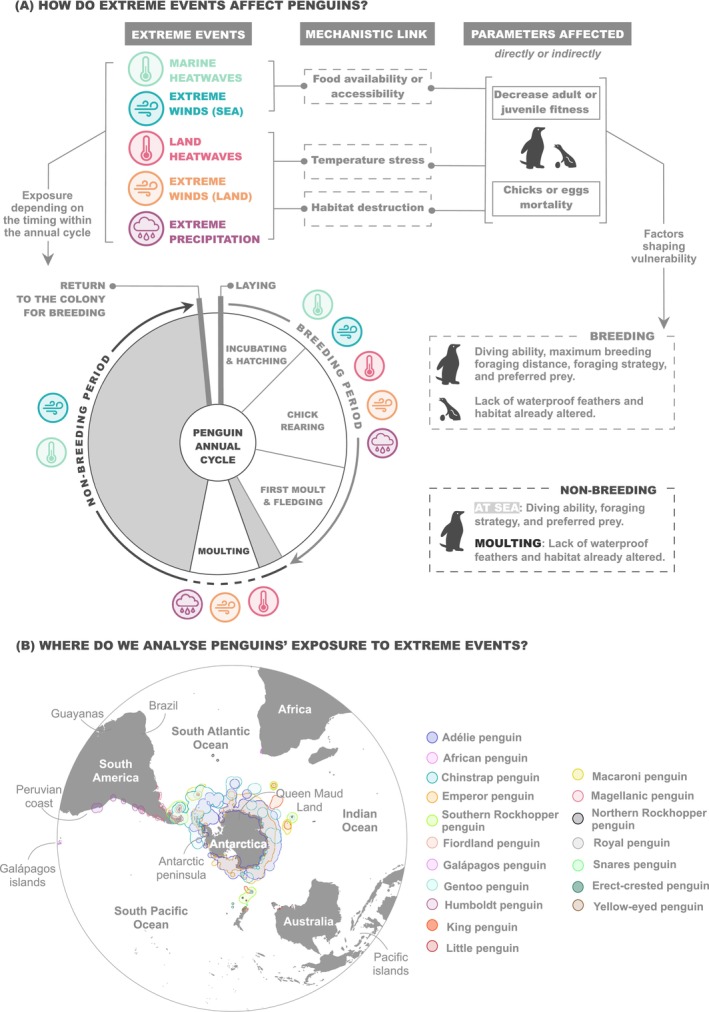
Impacts of extreme events and distribution hotspots of penguins. (A) Overview of how extreme events (marine heatwaves, extreme winds at sea, land heatwaves, extreme winds on land and extreme precipitation) affect penguins throughout their annual cycle. The timing and duration of each stage of the annual cycle vary among species, and the annual cycle of the Galápagos penguin differs substantially from the typical penguin cycle illustrated here; for a species‐specific annual cycle check Borboroglu and Boersma (2013). For a more in‐depth review on the topic, see Table S1. (B) Map showing the study area with the toponyms used throughout the manuscript and the hotspots for the 18 penguin species, modified from Gimeno et al. (2024).

Seabirds, and particularly penguins, are exposed to numerous climate and human‐driven stressors (Gimeno et al. 2024). They are also highly vulnerable to a range of extreme events throughout their life cycle, both on land and at sea (Chambers et al. 2015; Sydeman et al. 2012), although their overlap with extreme events has not been systematically quantified yet. Extreme events are likely to persist and intensify in the future (Lee et al. 2023), making it essential to study them separately from broader climate and human stressors. Due to their abrupt nature, extreme events can rapidly push species beyond their resilience thresholds in relatively short time scales. As a result, their impacts may be more severe than those caused by the gradual, long‐term shifts in the mean state driven by human‐induced climate change (Gruber et al. 2021). The effect of the extreme events will mainly depend on the life stage of penguins (adults, juveniles, chicks or eggs) and may occur through both direct and indirect mechanisms (Figure 1A and Table S1). For instance, heatwaves or extreme rainfalls may directly threaten survival, particularly of chicks and moulting penguins, which are more vulnerable to temperature stress due to their reduced insulation (Demongin et al. 2010; Ganendran et al. 2016). While indirect effects may arise from other extreme events, such as marine heatwave‐induced changes in productivity/food availability and accessibility, ultimately affecting the fitness of both juveniles and adults and leading to mortality in some cases (Morgenthaler et al. 2018). Additionally, these impacts can influence hatching and fledging success by reducing parental care (Berlincourt and Arnould 2015; Rebstock and Boersma 2018). Therefore, as air‐breathing central‐place foragers, penguins face land‐based extreme events during the moulting and breeding periods and, as top predators in marine food webs, they are also susceptible to the impacts of marine‐based extreme events (Woehler and Hobday 2023). Evaluating how extreme events overlap with critical penguin habitats is essential for identifying risks to these charismatic but vulnerable species. As recognised sentinels of marine ecosystems, the widely distributed penguins can also offer valuable global insights that can help pinpoint ecologically significant areas most at risk due to the multiple influences of extreme events (Boersma 2008; Hazen et al. 2019).

This study provides the first evaluation of the spatial overlap between multiple land‐ and sea‐based cumulative extreme events across the Southern Hemisphere, with a particular focus on existent significant areas, penguin hotspots, for the 18 penguin species (Figure 1B). We assess the spatial distribution and long‐term trends of various extreme events: marine heatwaves (MHWs), severe sea and land wind conditions (hereafter referred to as extreme winds), land heatwaves (HWs) and extreme precipitation on land across the Southern Hemisphere, selecting these event types based on their documented impacts in the literature (Table S1). This approach builds upon a well‐established methodology (Ramírez et al. 2017, 2021), incorporating the calculation of individual and cumulative extreme events in terms of intensity, duration (consecutive days) and frequency (days/year). Additionally, we also highlight areas expected to face significant risks in the future, based on long‐term time series (1993–2023). Therefore, we identify penguin species and their associated terrestrial and marine habitats that are most at risk from extreme events, providing insights into the perturbations that have, and will continue to, significantly impact key penguin distributions' hotspots. Overall, we present an adaptable framework (i.e., a scalable, spatially explicit approach that integrates multiple stressors and can be tailored to species or regions of concern) which can be continuously updated by extending the time series of environmental data and incorporating new assessments of additional extreme events as relevant data and insights become available.

## 2 Methods

### 2.1 Extreme Events Detection

We defined an extreme event as a discrete and prolonged episode in which environmental conditions exceed the seasonally varying 90th percentile for a duration of more than 5 days for a single marine variable (Sea Surface Temperature—SST, following Hobday et al. (2016)) and 3 days for the atmospheric variables such as land temperature, wind intensity and precipitation, following Gastineau and Soden (2009) and Perkins and Alexander (2013). The minimum threshold for duration may not capture short but biologically impactful events (e.g., Holt and Boersma 2022), yet ensures we are following the established climatological conventions for extreme events detection. We focused on the extreme events occurring over the last three decades. We therefore used a 30‐year baseline period (1993–2023) and detected extreme events within this timeframe. Since we are interested in the potential impacts of extreme events, trends were not removed before detecting them.

The analyses were conducted on a regular 1° grid for the whole Southern Hemisphere, extending up to 4° N to account for the entire distribution of the Galápagos penguin. The environmental data were downloaded with daily temporal resolution. The SST (°C) data used to detect marine heatwaves were downloaded from the National Oceanic and Atmospheric Administration (NOAA) ‘Optimum Interpolation Sea Surface Temperature’ (OI SST V2.1 (Huang et al. 2021); accessed January 2024). The original spatial resolution of this product is 0.25° and was interpolated onto 1° to match the spatial resolution of the atmospheric variables using bilinear interpolation. Atmospheric data were from the 5th generation of the European Centre for Medium‐Range Weather Forecasts (ECMWF) global climate and weather reanalysis data, known as ERA 5 (Hersbach et al. 2018) (accessed January 2024). This product combines model data with global observations using the laws of physics to create a comprehensive global dataset. Daily air temperature at 2 m (K converted to °C), wind intensity calculated from u and v wind components at 10 m (m/s), and total precipitation (m/h) data were used. Extreme events were detected using the *m_mhw* toolbox (Zhao and Marin 2019) in MATLAB version 23.2.0.2365128 (R2023b). This toolbox applies the definition of extreme events described in the previous paragraph. First, it calculates the percentile from the row environmental data, then identifies the days exceeding this percentile and aggregates consecutive extreme days. Finally, it keeps only those events that are equal to or greater than the minimum duration.

### 2.2 Cumulative Extreme Events: Mean and Trends

We considered intensity (°C for temperature, m/s for wind and m/h for precipitation), duration (consecutive days) and frequency (days/year) as metrics for extreme events because these are usually related to different effects on the population dynamics (González‐Trujillo et al. 2023). We calculated the mean, the trend over time, and its significance (slope and *p* value of the least‐square linear regression) for all three metrics on a per‐pixel basis in MATLAB. The post‐processing of all these outputs was performed in R version 4.3.1 (R Core Team 2023) using the following R packages: R.matlab v3.7.0 (Bengtsson 2022), tidyverse v2.0.0 (Wickham et al. 2019), sf v1.8.0 (Pebesma 2018), raster v3.6‐26 (Hijmans 2023), terra v1.7‐78 (Hijmans 2024), ggnewscale v0.4.10 (Campitelli 2024), colorspace v2.1‐0 (Zeileis et al. 2020).

**FIGURE 2:**
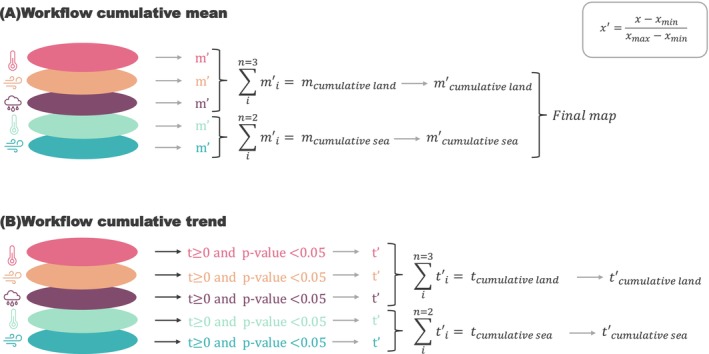
Illustrated workflow. (A) For the cumulative mean (m). (B) For the cumulative trend (t). In both workflows, each colour represents one type of extreme event: land heatwaves, extreme winds on land, precipitation, marine heatwaves, extreme winds at sea (from top to bottom). Each time we used the normalisation formula (top right) during the process, the arrow is in light grey. We followed these workflows to create a final map showing the cumulative spatial distribution for the intensity, duration, and frequency. See final maps at Figures 3 and 4.

The uneven distribution of cumulative extreme events was assessed using pixel‐based, equally weighted averages of intensity, duration and frequency across different extreme events (i.e., cumulative intensity mean). First, we normalised (Equation 1) the results for each variable. Then, we calculated the cumulative value for each metric and re‐normalised the final layer to sum up to one (Figure 2A illustrates the workflow). We also calculated the extreme event type that contributes the most to the total sum on a pixel basis and mapped the results.

(1) $$x^{\prime }=\frac{x-x_{\min}}{x_{\max}-x_{\min}}\;\text{with}\;0\leq x^{\prime }\leq 1$$

where *x*_min_ and *x*_max_ refer to the lowest and highest values of each variable, respectively.

To identify areas potentially at risk from cumulative extreme events in the future, we analysed past trends from 1993–2023 (per‐pixel slopes and significance) using the same workflow as for calculating the cumulative mean; therefore, we highlighted areas potentially at risk if trends are maintained in the future. However, we only kept pixels with positive and significant trends, as these are the trends most likely to negatively impact penguin species and their underlying ecosystems. Figure 2B illustrates this workflow. We also mapped the extreme event or the combination of extreme events that contribute to the cumulative value of the trends on a pixel basis, showing the type or types of extreme events with increasing trend according to the different regions.

### 2.3 Cumulative Extreme Events Within the Distribution Hotspots of Penguins

We downloaded the species distribution hotspots (i.e., areas that encompass the largest number of observations; therefore, potential Areas of Ecological Significance) for all the penguin species from the DigitalCSIC (https://doi.org/10.20350/digitalCSIC/16054). The authors calculated the hotspots using kernel density analysis based on occurrence data from GBIF (see procedure details in Gimeno et al. 2024). For the penguin species that had differentiated hotspots for the breeding and the non‐breeding season, we merged the hotspots to represent year‐round habitat use using the sf package in R.

We extracted the mean values of intensity, frequency and duration of extreme events for the pixels located within the penguins' hotspots. All the calculations were done considering extreme events on land and at sea separately. Pixels containing both sea and land habitats were treated according to the type of extreme event being analysed: they were considered as sea pixels when calculating extreme events at sea and as land pixels when calculating land‐based extreme events. For the Northern Rockhopper penguin ( *Eudyptes moseleyi* ), we were not able to extract extreme events on land because the islands inhabited by this species are too small to be resolved at 1° grid resolution (download from: https://www.naturalearthdata.com/downloads/50m‐physical‐vectors/). Then, we calculated the mean value for each extreme event type and summed these to obtain the total cumulative extreme event values for each penguin species (the maximum and minimum values are shown in Figure 5A). To facilitate comparison among penguin species, these cumulative values were normalised by the largest mean cumulative value obtained over all hotspots. Therefore, final values range from 0 to 1, indicating the species least and most exposed to cumulative extreme events (Figure 5B, lolliplots). Finally, we assess the relative contribution of each extreme event to the total mean cumulative sum (stacked bars in Figure 5B).

For the trends, we extracted the values of the pixels with positive and significant trends within the penguins' hotspots, and for all the calculations, we considered extreme events on land and at sea separately. Then, we calculated the mean value for each extreme event type, and we summed all the mean values to have the total cumulative extreme events trend values for each penguin species (the maximum and minimum values are shown in Figure 6A, with the corresponding species indicated by letters ‘S’ for sea‐ and ‘L’ for land‐based extreme events in Figure 6B). Since only positive and significant trends were retained, and the area of potential exposure varies greatly among penguin species, we divided the mean value of each extreme event type by the number of pixels within each species' hotspot. This adjustment reduces the estimated mean values for species less exposed to such trends. To provide insights into how representative the trends were within each hotspot, we calculated the percentage of pixels within the hotspot covered with positive and significant trends (lollipops in Figure 6B). Finally, we assessed the relative contribution to the sum of each extreme event to the total mean cumulative trend (stacked bars in Figure 6B).

We organised the results for each penguin species, both the mean values and the trends, based on the IUCN Red List categories (https://www.iucnredlist.org, accessed January 2025). The IUCN gathers data from experts and applies a set of quantitative, standardised criteria, such as population size, trends and geographic range, to classify species. We included this classification in our plot because it not only indicates each species' endangered status but also serves as an indicator of their potential vulnerability to increasing cumulative pressures.

## 3 Results

### 3.1 Characteristics of Extreme Events in Recent Decades (1993–2023)

**FIGURE 3:**
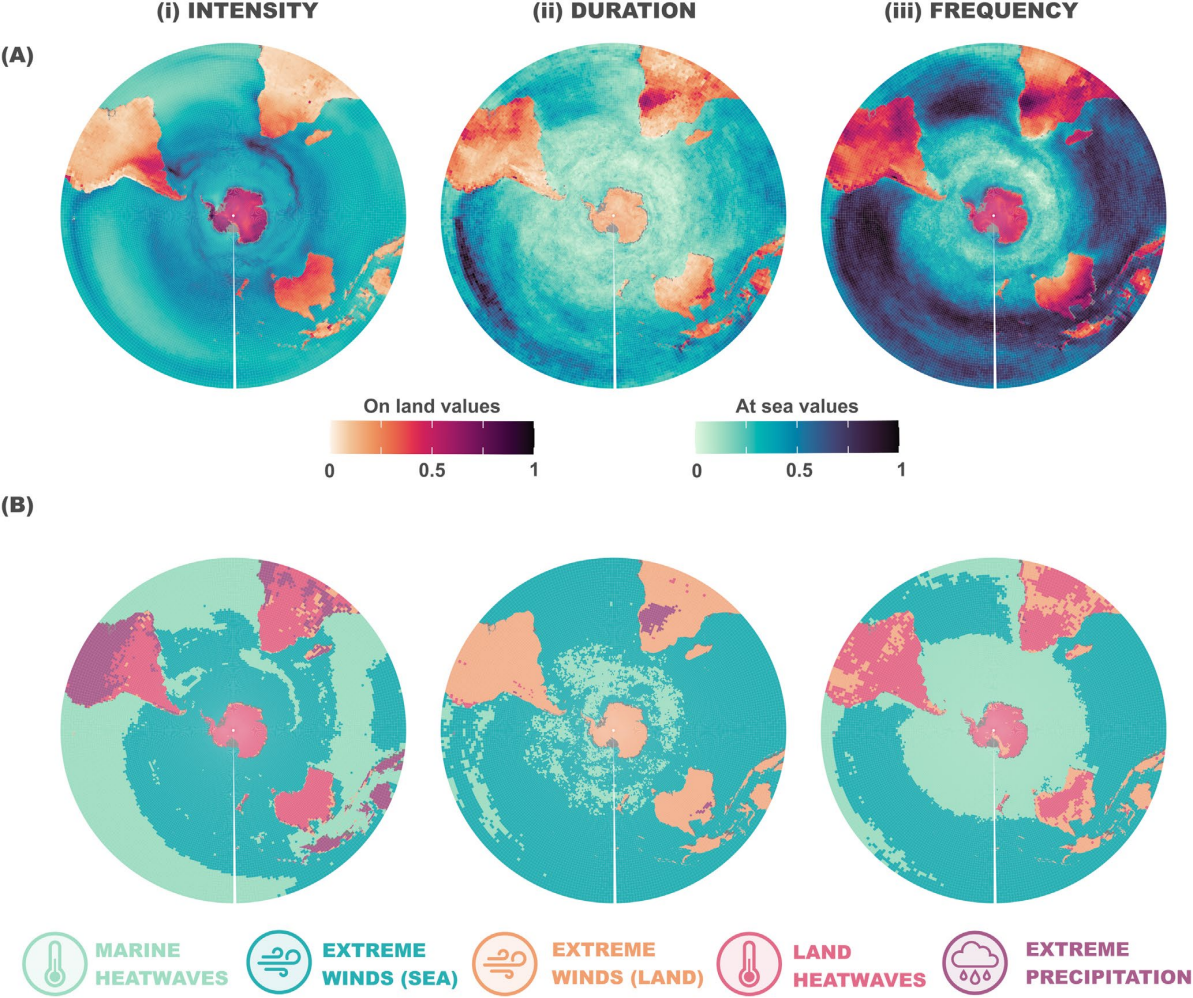
Cumulative mean of extreme events by pixel. (A) Maps of the magnitude of the cumulative mean (indicated by respective colour scale bars for land and sea) of all the types of extreme events calculated according to (i) intensity, (ii) duration, and (iii) frequency. (B) Maps depicting the extreme event with the greatest contribution by pixel (extreme event indicated by the colour of the symbols below), categorised by (i) intensity, (ii) duration, and (iii) frequency. Grey pixels represent a lack of data for environmental variables.

Using intensities, durations and frequencies detected on a pixel basis for the various extreme events (details in methods and Figure S1), we assessed the mean distribution of the cumulative magnitude of these metrics over the last three decades (1993–2023) using a spatially explicit approach. Our assessments revealed that the highest mean cumulative intensities on land occurred from mid to high latitudes (Figure 3Ai). The extreme event contributing most to cumulative intensity values varied by region: HWs predominated from mid to high latitudes, while extreme precipitation was the main type of extreme event at low latitudes (Figure 3Bi). At sea, the large cumulative intensity values occurred from mid to high latitudes (Figure 3Ai). The MHW was the main contributor to cumulative intensity from mid to low latitudes, while extreme winds at sea dominated from mid to high latitudes. A few exceptions to this general pattern were observed in the equatorial part of the Indian Ocean and above the Pacific Islands (Figure 3Bi). Frontal regions such as the Sub‐Tropical and the Sub‐Antarctic fronts in the Atlantic and Indian Oceans emerged as areas with the highest cumulative intensity, primarily driven by intense MHWs (Figure 3Ai,Bi).

Long‐lasting extreme events on land extended from mid to low latitudes (Figure 3Aii). Extreme winds were the primary contributors to values of cumulative duration on land, except in southwestern Africa, where extreme precipitation predominated (Figure 3Bii). At sea, the highest values of cumulative duration occurred in the Central and East Pacific (Figure 3Aii). Overall, extreme winds were the main contributor to cumulative duration values across the Southern Hemisphere. However, in those regions with the highest and lowest cumulative durations, MHWs had a greater contribution (Figure 3Bii).

The highest cumulative frequency values across the entire Southern Hemisphere occurred in the mid to low latitudes (Figure 3Aiii). On land, the highest values were observed in Central Brazil, Central Africa, Northern Australia and Antarctica (Figure 3Biii). HWs were the main contributor to cumulative frequency values on land over the Southern Hemisphere, except for some mid to low latitude regions where extreme winds emerged as the main contributor (Figure 3Biii). At sea, MHWs were the main contributor to cumulative frequency from mid to high latitudes, the equatorial part of the South Pacific Ocean and the eastern boundary upwelling systems. In the other regions, extreme winds dominated the cumulative frequency (Figure 3Biii).

### 3.2 Characteristics of Extreme Events Trends in Recent Decades (1993–2023)

Using annual mean intensities, durations and frequencies for the various extreme events, we assessed the long‐term (1993–2023) trends by estimating the slopes and significances of pixel‐based, least‐squares linear regressions using a spatially explicit approach (details in methods and Figure S2). The long‐term trends of individual extreme events were unevenly distributed spatially, with frequencies showing relatively uniform spatial patterns, while trends in intensity and duration lacked spatial consistency (Figure S2).

**FIGURE 4:**
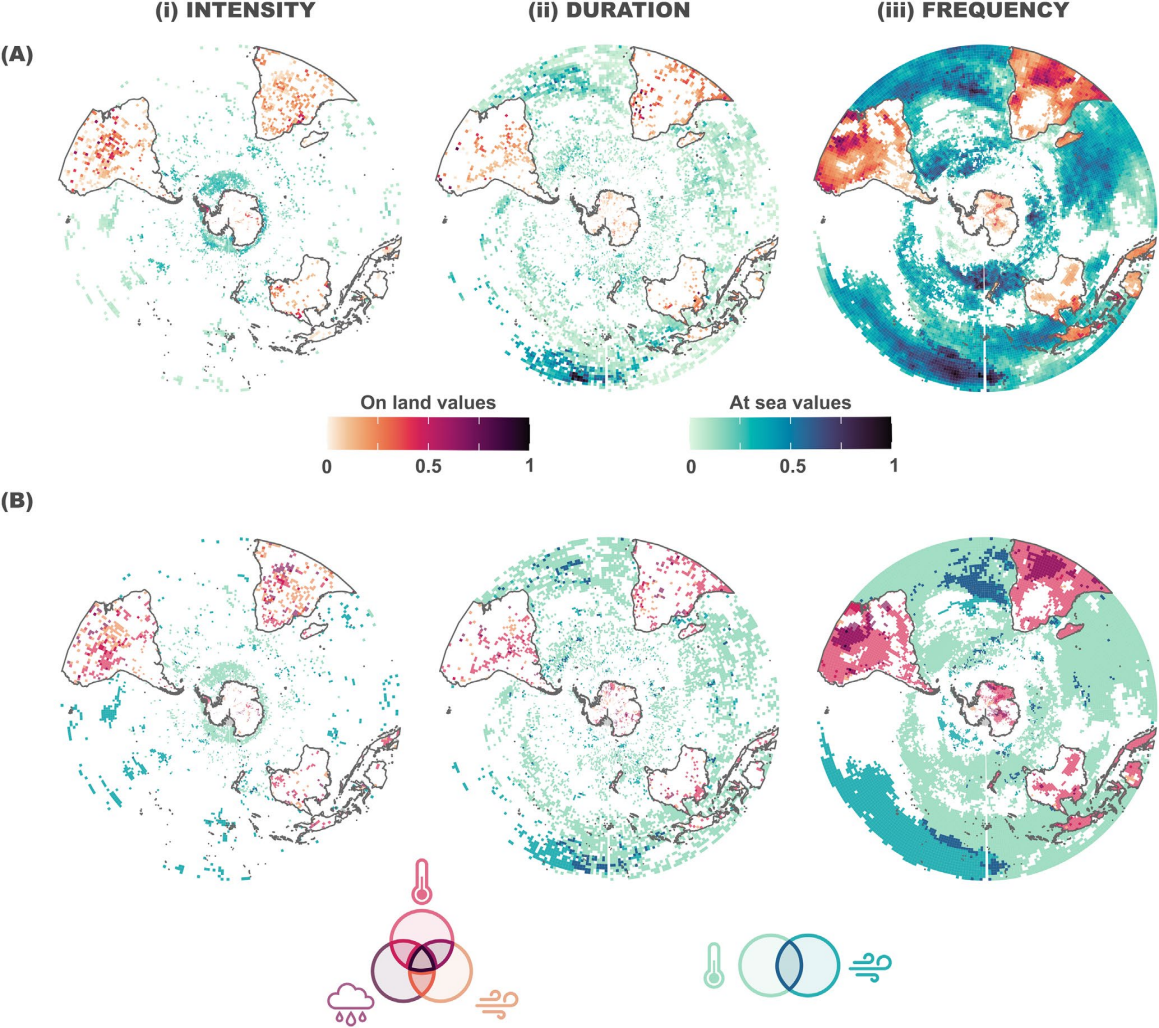
Cumulative trends of extreme events by pixel. (A) Maps of the cumulative magnitude of the positive and significant trends (indicated by respective colour scale bars for land and sea) for all the types of extreme events calculated according to (i) intensity, (ii) duration, and (iii) frequency. (B) Maps depicting the type or types of extreme event with positive and significant trend by pixel (extreme event indicated by the colour of the symbols below), categorised by (i) intensity, (ii) duration, and (iii) frequency. Grey pixels represent a lack of data for environmental variable.

Positive and significant cumulative intensity trends extended 13% of the total land area, mainly from mid to low latitudes, as well as the Antarctic Peninsula (Figure 4Ai). Of this area, 7% showed overlap of multiple extreme events, while the remaining 6% was dominated by a single extreme event. Within the 7% affected by multiple extreme events, 4% corresponded to the spatial overlap of HWs and extreme winds, 2% of extreme precipitation and extreme winds, and 1% of precipitation and HWs (Figure 4Bi). At sea, positive and significant trends in intensity covered 18% of the total ocean area (Figure 4Bi). However, trends in MHWs and extreme winds were spatially segregated, with MHWs primarily concentrated around Antarctica and extreme winds occurring mainly in mid to low latitudes. Therefore, only 1% of the total ocean area experienced positive and significant trends overlap between MHWs and extreme winds trends.

For duration, the largest positive and significant trends were primarily found from mid to low latitudes. On land, these trends covered 16% of the total area, with 3% showing spatial overlap of HWs and extreme precipitation, 2% of HWs and extreme winds and 1% of precipitation and winds (Figure 4Bii). At sea, positive and significant duration trends covered 27% of the total ocean area and were particularly strong over the Western Pacific. Of this area, 3% presented the co‐occurrence of MHWs and extreme winds (mainly in the north‐westernmost regions of the South Pacific Ocean) (Figure 4Bii).

Positive and significant frequency trends covered 44% of the total land area, including most of South America and Africa, with the highest cumulative trends observed in the Northeast of South America and central Africa (Figure 4Aiii). East Antarctica and the Antarctic Peninsula, some regions of Australia, and the Pacific Islands also showed significant and positive trends in frequency. Of the total impacted area, 19% corresponded to cumulative trends; 1% of this area has a spatial overlap of the three extreme events, 9% showed an overlap of extreme precipitation and HWs, 8% of HWs and extreme winds, and 1% of extreme winds and extreme precipitation (Figure 4Biii). The largest positive cumulative trends observed in the Northeast of South America corresponded to the combined effect of winds and HWs. Positive and significant frequency trends covered 50% of the total ocean area in the Southern Hemisphere. MHWs explain the majority (45%) of the observed positive cumulative trend in frequency. Only 5% of the region presenting positive cumulative trends in frequency was under the influence of MHWs and extreme winds. This was particularly the case in the eastern South Atlantic Ocean, where large cumulative frequency trends were observed. Large cumulative trends were also observed in the Western Pacific. In this case, winds were the extreme event leading to the large cumulative trends in frequency.

### 3.3 Extreme Events Within the Distribution Hotspots of Penguins

**FIGURE 5:**
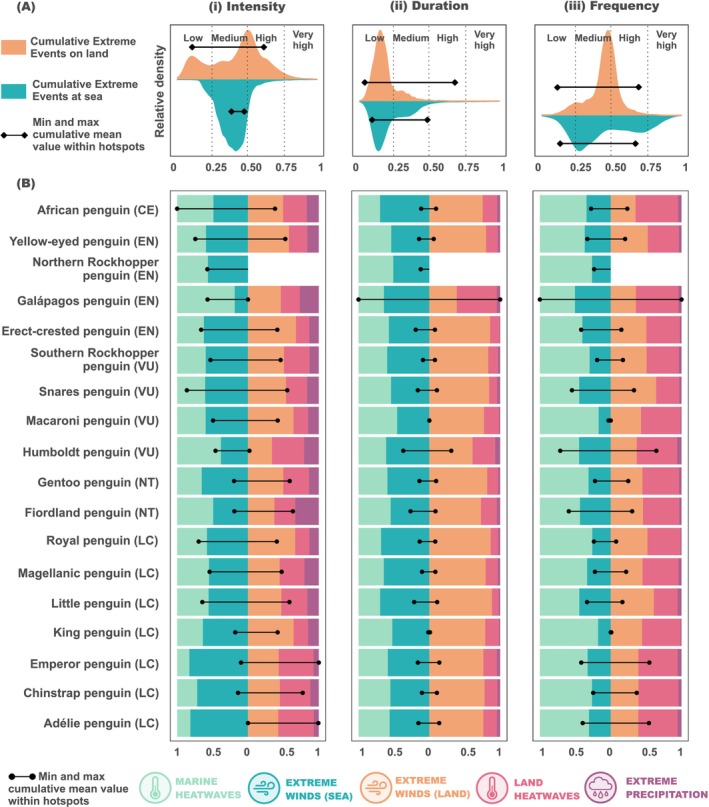
Extreme events within the distribution hotspots of penguins. (A) The density plot shows the distribution of cumulative extreme event values on land and at sea across the Southern Hemisphere, along with the maximum and minimum mean cumulative values observed within penguin hotspots. The *y*‐axis represents relative density, but exact values are not displayed, as the primary goal is to highlight which values are more frequent. (B) The lollipops show the magnitude of the mean cumulative extreme event within each penguin hotspot. The values were normalised according to the maximum cumulative value obtained for each metric, considering all the penguin species distinguishing sea (left) and land values (right) (Adélie value for intensity of land cumulative extreme events, African penguin values for intensity of sea cumulative extreme events, and Galápagos value for duration and frequency of land and sea extreme events). The stacked bars show the relative contribution of each type of extreme event within the hotspot, distinguishing sea (left) and land values (right). Next to each penguin name, there is the IUCN status for each penguin species: CR, Critically Endangered; EN, Endangered; VU, Vulnerable; NT, Near Threatened; LC, Least Concern. For all the plots, we show the results according to (i) intensity, (ii) duration, and (iii) frequency.

Within penguin hotspots, the different cumulative metrics presented mean values that rank from low (0–0.25) to high (0.5–0.75), showing how different penguin species were exposed to highly heterogeneous environmental conditions, both within their hotspot and in comparison to the entire Southern Hemisphere context (Figure 5A). Only the values for the cumulative intensity and duration at sea are consistently ranked as medium (values from 0.25 to 0.50) for all penguin species (Figure 5A).

When looking at the values within the penguins' hotspots, our analysis showed that the African ( *Spheniscus demersus* ) and Snares ( *Eudyptes robustus* ) penguins were the species with the highest intensity values of cumulative extreme events at sea (Figure 5Bi). On land, Antarctic species such as the Adélie ( *Pygoscelis adeliae* ) and Emperor ( *Aptenodytes forsteri* ) penguins were the ones facing the highest intensity of cumulative extreme events (Figure 5Bi). Galápagos penguins ( *Spheniscus mendiculus* ) faced the longest and most frequent extreme events both at sea and on land (Figure 5Bii,iii). Compared to the rest of the Southern Hemisphere, these values correspond to high durations and frequencies, with land‐based events nearing very high levels in the case of Galápagos penguins (values from 0.75 to 1; Figure 5Aii,iii).

When evaluating the relative contributions of the different extreme events to cumulative intensity values within the penguins' distribution hotspots, we observed contrasting patterns across penguin species (Figure 5Bi). At sea, extreme winds were the main contributor for almost all the species' cumulative intensity (14 out of the 18 penguin species). On land, extreme wind intensity was also the main contributor for 12 penguin species (relative contributions > 0.5 for six of them), while HW predominates in four of them (relative contributions > 0.5 for one of them). Extreme precipitation was the dominant extreme event cumulative intensity for the Fiordland penguin ( *Eudyptes pachyrhynchus* , relative contributions > 0.3). The percentage of contribution in duration was dominated (relative contributions > 0.5) by extreme wind at sea and on land for almost all the species (Figure 5Bii). The exceptions were the Macaroni penguin ( *Eudyptes chrysolophus* ) at sea and the Galápagos penguin on land. In these species, MHW and HW were the predominant extreme events in terms of duration, respectively. Regarding the contribution of the different extreme events to the frequency, MHWs and HWs were the predominant extreme events (Figure 5Biii). At sea, the only exception was the Galápagos penguin, which was mostly exposed to extreme winds. On land, five species have extreme winds as the predominant extreme event. Precipitation had barely any contribution to the cumulative mean for duration and frequency.

### 3.4 Extreme Events Trends Within the Distribution Hotspots of Penguins

**FIGURE 6:**
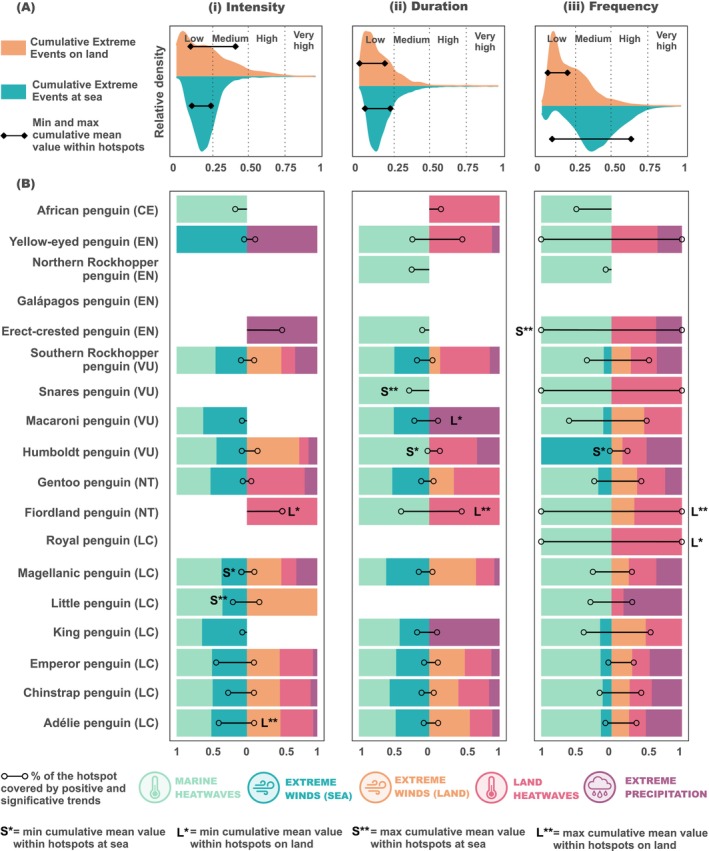
Extreme events trends within the distribution hotspots of penguins. (A) The density plot shows the distribution of cumulative trend extreme events values on land and at sea across the Southern Hemisphere, along with the maximum and minimum mean cumulative trend values observed within penguin hotspots. The *y*‐axis represents relative density, but exact values are not displayed, as the primary goal is to highlight which values are more frequent. (B) The lollipop shows the percentage of the pixels of the hotspot covered by significant and positive trends of extreme events. The stacked bars show the relative contribution of positive and significant trends for each type of extreme event within the hotspot. The letters indicate the penguin species with the lowest (*) and the highest (**) cumulative trend value (marked in A). Next to each penguin name, there is the IUCN status for each penguin species: CR, Critically Endangered; EN, Endangered; VU, Vulnerable; NT, Near Threatened; LC, Least Concern. For all the plots, we show the results according to (i) intensity, (ii) duration, and (iii) frequency.

Within their distributions' hotspots, penguins exhibited cumulative trend mean values that were mainly categorised as low (values from 0 to 0.25) relative to the rest of the Southern Hemisphere. The only exceptions were land intensity, classified as medium (values from 0.25 to 0.5), and sea frequency, classified as high (values from 0.5 to 0.75) (Figure 6A). All the penguin species, except the Galápagos penguin, showed positive and significant trends during the last three decades for at least one type of extreme event (Figure 6B). The percentage of coverage of these trends within the hotspots depended on the metric evaluated, the species and the environment (i.e., at sea or on land). The highest coverage values were observed in frequency, with Erect‐crested, Snares, Fiordland and Royal penguins (Figure 6Biii).

Only Southern Rockhopper ( *Eudyptes chrysocome* ), Emperor, Chinstrap ( *Pygoscelis antarcticus* ) and Adélie penguins exhibited positive and significant trends for all the metrics and types of extreme events within their hotspots. Among the three extreme event metrics, frequency was the most prevalent within penguin hotspots, with the Galápagos penguin being the only exception, showing no trends. This was followed by duration and, finally, intensity. This pattern aligns with the broader trend observed across the Southern Hemisphere. Among the various types of extreme events, MHWs and HWs showed the most widespread increasing trends within penguins' hotspots.

The contributions of different types of extreme events to the cumulative trends differ from those observed in the cumulative mean (Figures 5B and 6B). Overall, the wind contribution decreased. In contrast, precipitation, which had little contribution to the mean cumulative value, emerged as the only contributor on land for some species. Notably, it showed increased intensity for Yellow‐eyed and the Erect‐crested penguins, as well as the duration for Macaroni and the King ( *Aptenodytes patagonicus* ) penguins (Figures 5Bi and 6Bi). For frequency, the cumulative trend at sea was dominated by MHWs except for the Humboldt penguin ( *Spheniscus humboldti* ) (Figures 5Biii and 6Biii).

## 4 Discussion

This study presents the first highly detailed, large‐scale spatial assessment of the overlap between penguins' distributions and terrestrial and marine extreme events (MHWs, extreme winds, HWs and extreme precipitation). By examining values within penguins' hotspots distribution to both individual and cumulative extreme events, we offer insights into the potential risks these events may pose to these emblematic species, as well as to the areas they inhabit in the Southern Hemisphere. Based on observed trends over recent decades, extreme events are expected to increase in frequency and severity in the future, likely presenting significant challenges for penguins and their ecosystems. Our analysis highlighted that, among the 18 penguin species, African, Snares, Emperor, Adélie and Galápagos penguins experienced the highest cumulative values of extreme events. Additionally, we identified that all the penguin species, except the Galápagos penguin, showed trends in their hotspots that suggest a potential increase in the intensity, duration and frequency of extreme events.

### 4.1 Global Patterns of Extreme Events in Areas of Ecological Relevance of the Southern Hemisphere

At sea, the regions presenting the largest mean cumulative intensity values of extreme events were observed along the Sub‐Tropical and the Sub‐Antarctic fronts in the Atlantic and Indian Oceans. Oceanic fronts are known to be highly productive areas that provide predictable feeding conditions for marine predators and many other marine species (Bost et al. 2009; Reisinger et al. 2018). The high‐intensity values observed in these areas were mainly explained by MHWs (Figure 3). The MHWs in these areas are marked by high intensity and frequency but not by prolonged duration (Figure S1). These characteristics are primarily driven by frontal displacements (Artana, Rodrigues, et al. 2024; Holbrook et al. 2019). The Central‐East Pacific upwelling region exhibited one of the largest cumulative values of long and frequent extreme events, due to MHWs (Figure 3). In the tropical Pacific, MHWs are usually associated with El Niño‐Southern Oscillation (ENSO) events, which explain the long durations observed (Holbrook et al. 2019; Sen Gupta et al. 2020). Beyond their local effects, both El Niño and La Niña events can remotely drive the development of MHWs in other regions through atmospheric and oceanic teleconnections (e.g., Sen Gupta et al. 2020; Artana, Rodrigues, et al. 2024). Consequently, ENSO may influence species in different ways across regions. MHWs can impact marine ecosystems by reducing primary productivity; however, this effect varies with latitude, showing the inverse trend in the southernmost regions (Fernández‐Barba et al. 2024; Sen Gupta et al. 2020). Gaining further insights into the relationship between MHWs and primary production, together with the phytoplankton community, is especially relevant to understanding and predicting their actual impacts on the marine ecosystems (Li et al. 2024).

Continental Antarctica stood out as one of the regions most exposed to intense and frequent land‐based extreme events. Patagonia, South Africa, Australia and New Zealand were also highly exposed to the cumulative intensity of land‐based extreme events. The Galápagos Islands and the Peruvian coast showed particularly high values of cumulative duration and frequency of these events. All these coastal areas are well‐known areas of ecological relevance due to their rich biodiversity (Ramírez et al. 2017). In all these regions, HW contributed most significantly to the total cumulative values, aligning with the ongoing climate change scenario (Harris et al. 2018; IPCC 2021). Heatwaves can be associated with droughts or fires (Sutanto et al. 2020), but also with water pulses from ice melting or rainfalls in polar deserts (Nielsen et al. 2012). In both cases, these events lead to habitat degradation, which is particularly threatening for terrestrial breeding marine predators (Sojitra et al. 2024). Based on estimated trends for extreme events over the past few decades, and assuming these trends will continue, we anticipated an increase in the intensity and frequency of HW and extreme precipitation in the Antarctic Peninsula. Recent studies highlight the potential impact of increasing temperatures on vegetation colonisation in the region (Roland et al. 2024), with vegetation changes also influenced by penguin colonies through nutrient enrichment, trampling or guano accumulation. However, further research is needed to understand how these trends will evolve with the growing frequency of extreme events and the effect of these changes on ecosystem functioning.

### 4.2 Patterns of Extreme Events in Distribution Hotspots of Penguins

Extreme events can impact population dynamics and organisms in different ways, with their effects often linked to the specific metrics of the events, such as intensity, duration and frequency, but also with the unique characteristics of the species (González‐Trujillo et al. 2023). For instance, already threatened species or species with a restricted distribution may be particularly vulnerable to extreme events. Our assessments revealed that the predominant extreme event affecting penguins varied depending on both the metric considered and the species examined. Extreme winds were the primary contributors to cumulative intensity and duration for most of the penguins' distribution hotspots, whereas MHW and HW posed the greatest contribution to the cumulative frequency at sea and on land, respectively. Despite this general pattern, the uneven distribution of extreme events implies that different species, and even populations within the same species, may be facing contrasting extreme events.

The African penguin and the Snares penguin faced the highest cumulative values of intense extreme events at sea (Figure 5). However, these values are classified as medium compared to the rest of the Southern Hemisphere, and no mass mortality caused by extreme events has been documented for these species. Despite this lack of reported effects, the African penguin is currently the most endangered penguin species, undergoing an alarmingly rapid population decline, likely driven by a reduction in food availability (Crawford et al. 2022). While extreme events have not been highlighted as a primary threat to this species (BirdLife International 2024), extreme events at sea may exacerbate the challenges faced by already vulnerable populations. In particular, extreme water temperatures associated with MHW, which accounted for half of this species' value of cumulative intensity of extreme event, can alter prey distribution and reduce its accessibility to penguins (Van Eeden et al. 2016). Moreover, penguins' inability to fly limits their ability to search for food compared with other marine seabirds, amplifying the impact of local prey disruptions (Woehler and Hobday 2023). The Snares penguin maintains a stable population due to continued prey availability (Mattern et al. 2009). However, its restricted distribution makes it more potentially vulnerable to localised threats such as fisheries, introduced predators, and climate change, with extreme events potentially exacerbating these risks (Mattern et al. 2009).

The Antarctic Adélie and Emperor penguins were exposed to the highest cumulative intensity values of land‐based extreme events among all penguin species (Figure 5). Events with high intensity values are usually linked with abrupt mass mortalities (González‐Trujillo et al. 2023). The intensity values for these two species were notably high compared to the rest of the Southern Hemisphere, likely reflecting the documented impacts of such extreme events on these species (Fretwell et al. 2023; Fretwell and Trathan 2019; Ropert‐Coudert et al. 2018, 2015). Similar impacts have also been observed in other Antarctic seabirds (Descamps et al. 2023). These impacts include massive breeding failures coinciding with changes in the sea ice configuration due to extreme winds (Fretwell and Trathan 2019; Ropert‐Coudert et al. 2018), intense snowfalls (Descamps et al. 2023) or unusual warm temperatures driving unprecedented rain episodes and snowmelt (Ropert‐Coudert et al. 2015). However, in most cases, these breeding failures were likely the result of the cumulative effect of multiple threats rather than a single one (Ropert‐Coudert et al. 2015). Although isolated breeding failures in long‐lived species like penguins may have a relatively minor impact on population trends, the observed trends in the frequency of extreme events in Antarctica could lead to more frequent breeding failures, posing significant challenges for these species (Harris et al. 2018). This could compound other growing pressures, such as increasing human presence in Antarctica, whose impact on the ecosystem remains uncertain (Tejedo et al. 2022). Although Adélie and Emperor penguins face some of the highest intensities of land‐based extreme events, they may also exhibit adaptive responses such as colony relocation, which we discuss further below.

The Galápagos penguin experienced the highest cumulative extreme event duration and frequency in both its marine foraging areas and terrestrial breeding sites compared with other penguin species, and these values were also within the high cumulative values observed across the Southern Hemisphere (Figure 5). Prolonged extreme events have been associated with reduced survival and recruitment rates, while an increased frequency of extreme events is linked to decreased birth rates and diminished recruitment (González‐Trujillo et al. 2023). In regions subjected to both prolonged and frequent extreme events, this can trigger phenological changes to the species inhabiting those ecosystems (González‐Trujillo et al. 2023). The increasing frequency of such events may further hinder the Galápagos penguin's ability to recover to previous population numbers (Vargas et al. 2006). Moreover, its low genetic diversity makes Galápagos penguins particularly vulnerable to emerging land‐based threats, such as mosquito‐borne diseases and parasites, whose incidence may be heightened by the more favourable environmental conditions for mosquitoes resulting from the predicted increase in temperatures and rainfall (Arauco‐Shapiro et al. 2020; Dueñas et al. 2021). Nevertheless, the Galápagos penguin's phenological flexibility, discussed below, may buffer some of these impacts.

Little and Yellow‐eyed penguins were also exposed to high intensity values of cumulative extreme events both at sea and on land (Figure 5). Previous research has noted the vulnerability of these species to extreme events at sea (Cannell et al. 2023; Mattern et al. 2017; Saraux et al. 2016). On land, direct effects from extreme events include adult mortality during the moult because of heat stress (Ganendran et al. 2016; Mattern et al. 2017). Indirectly, extreme events can impact these species through habitat degradation, which can exacerbate other threats and has likely contributed to the decline of Little penguins in Tasmania (Stevenson and Woehler 2007) and reduced the breeding success of the Yellow‐eyed penguin (Clark 2007).

In addition to the high values of cumulative mean currently observed in certain penguin hotspots, nearly all penguin species faced increasing trends of the extreme events in their breeding areas and foraging grounds over the last decades, with the notable exception of the Galápagos penguin. If current climate trends continue, most penguin species will likely become even more vulnerable to extreme events. Indeed, many penguins have already shown adverse thermoregulatory impacts due to HW and extreme precipitation (Demongin et al. 2010; Holt and Boersma 2022), as well as increased adult and chick mortality caused by extreme storms and rainfall (Wolfaardt et al. 2012). Additionally, unusual moulting patterns and mortality linked to harsh oceanic conditions have been reported in these species (Morgenthaler et al. 2018). However, not all penguin species have shown clear links between extreme events and population fluctuations in previous studies. This may be due to the limited impact of these extreme events on certain species or populations, or because the die‐offs associated with these events are more often reported in news outlets than in scientific literature (Holt and Boersma 2022).

Building on the examples above, some penguin species exhibit behavioural plasticity and phenological adaptations that may confer resilience to extreme events. For instance, Little penguins are known for their flexible diets and foraging distributions (Cullen et al. 1991; Flemming et al. 2013), which enable them to respond to variable prey availability caused by extreme events at sea. On land, Emperor penguins may adapt to extreme events by adjusting breeding habitat or by relocating between colonies (Kooyman and Ponganis 2017). Also, the phenological flexibility exhibited by the Galápagos penguin may have emerged as an adaptation to the high environmental variability typical of its region (Boersma 1978). In addition, this species shows a unique behaviour among penguins, as parents continue to feed their young after fledging (Boersma et al. 2017). This environmental variability is also captured in our results, as no trends in extreme events were detected within the Galápagos penguin's hotspot. Consequently, this flexibility allows the species to adapt to highly heterogeneous and variable environmental conditions by selecting optimal periods for these critical life history events. This strategy may help mitigate the adverse impacts of land‐based extreme events on breeding success and adult survival (Boersma 1978; Boersma et al. 2017). Nevertheless, the adaptive capacity exhibited by this species is not limitless, and certain extreme marine events, particularly those associated with ENSO events, have had severe population‐level impacts (Vargas et al. 2006). This limited capacity to adapt also extended to other adaptations, like the geographically limited conditions for colony relocation for Emperor penguins. Therefore, even if some penguin species may remain relatively insensitive to extreme events below certain thresholds of intensity, duration or frequency, we anticipate that once these thresholds are surpassed, penguin populations could experience significant, and potentially irreversible, responses. Moreover, the cumulative effects of multiple extreme events over short time periods will prevent any type of population recovery or adaptation.

### 4.3 Further Steps and Conservation Implications

Despite the insights offered by this study, several limitations must be acknowledged. Here, we consider the cumulative spatial overlap of various extreme events occurring both on land and at sea; further studies should additionally consider the synchronicity among the different types of extreme events, as spatial overlap does not necessarily imply temporal coincidence. Furthermore, beyond studying cumulative extreme events solely at the ocean surface, future studies could incorporate the depth component that, in some phenomena like MHWs, can act as a driver of food availability (Großelindemann et al. 2022). Future analyses could also assess more variables at sea, like ocean acidification or oxygen loss (Gruber et al. 2021). Finally, investigating the synergies between extreme events and other stressors (e.g., fishing pressure), along with species‐specific vulnerabilities to these cumulative impacts, could provide a more comprehensive risk assessment.

Assessing species‐specific vulnerabilities requires establishing and maintaining long‐term monitoring programmes to track the effects of extreme events on penguins and their habitats. Integrating detailed demographic data into the analysis can directly link these extreme events to population outcomes, and focusing on individual species will allow for more precise temporal analyses, particularly in identifying overlaps between extreme events and critical life stages, such as moulting and breeding seasons. However, this level of detail was beyond the scope of this study due to the huge variability in the timing and duration of these life‐cycle stages across species.

Our results underscore the rising frequency of cumulative extreme events, revealing a consistent and significant trend across multiple regions of the Southern Hemisphere, both at sea and on land, and within penguins' hotspots. These trends should be considered in management strategies, as they represent a significant threat to penguins, especially species with limited recovery capacity or those with small populations and restricted distributions (Frederiksen et al. 2008; González‐Trujillo et al. 2023; Sepúlveda et al. 2020). The approach we propose involves three main steps: (1) Identify priority hotspots, areas relevant for penguins where cumulative extreme event values are high and/or trends are steeply increasing. (2) Consider local stressors, as the pressures of extreme events may be compounded by growing human activity, even where the ecological consequences remain uncertain (Tejedo et al. 2022). (3) Implement adaptive management actions, for example, in Antarctica, our findings support the need for strengthened environmental protections under the Antarctic Treaty System, particularly in areas where increasing extreme events coincide with increasing human activities. At sea, strategies to mitigate marine heatwaves should include fisheries regulations and marine spatial planning. On land, efforts to preserve and restore breeding habitats can be effective local actions to enhance species resilience to heatwaves and extreme precipitation. This approach is iterative, allowing actions to be updated as monitoring data and climate projections evolve, or other drivers of environmental change emerge. Importantly, the specific suite of solutions should be evaluated at regional scales, focusing on the species and areas identified as most vulnerable by our analyses, ensuring that interventions are targeted, efficient, and maximise conservation impact. These measures can serve as practical management solutions while the international community works toward addressing the ongoing climate crisis.

## Author Contributions

**Míriam Gimeno:** conceptualization, formal analysis, methodology, visualization, writing – original draft. **Andre Chiaradia:** supervision, writing – review and editing. **Marta Coll:** funding acquisition, supervision, writing – review and editing. **Francisco Ramírez:** conceptualization, funding acquisition, supervision, writing – review and editing. **Camila Artana:** conceptualization, formal analysis, methodology, supervision, writing – review and editing.

## Conflicts of Interest

The authors declare no conflicts of interest.

## Supporting information


**Data S1:** gcb70562‐sup‐0001‐Supinfo.docx.

## Data Availability

The results and the code that support the findings of this study are available in DigitalCSIC: https://doi.org/10.20350/digitalCSIC/17541. These data were derived from the following resources available in the public domain: NOAA: https://www.ncei.noaa.gov/products/optimum‐interpolation‐sst; Copernicus https://cds.climate.copernicus.eu/datasets/reanalysis‐era5‐single‐levels?tab=overview.
