# Supplementary material for: Cumulative Extreme Events Threaten Penguin Habitats Across the Southern Hemisphere
Source: Glob Chang Biol. 2025 Oct 23;31(10):e70562. doi: 10.1111/gcb.70562 (PMC12547633; doi:10.1111/gcb.70562)
Supplement: Supplementary file 1 — Data S1: gcb70562‐sup‐0001‐Supinfo.docx. [file GCB-31-e70562-s001.docx]

**Supplementary Material**

**Brief Description of the Supplementary Figures**

**Fig. S1. Mean values for the different extreme events and metrics:** MHWs are particularly intense, frequent, and long lasting along the equatorial Pacific (Fig. S1A). They are also especially intense (>4°C) along the subtropical front and the Agulhas Current in the South Atlantic. Intense extreme storms at sea are located from mid to high latitudes (Fig. S1B i). In contrast, extreme storms’ duration and frequency exhibit an inverse pattern than the intensity, with higher values from mid to low latitudes (Fig. S1B ii and iii). HWs present a clear pattern of higher intensity from mid to high latitudes with the highest intensities in Antarctica (>10ºC; Fig. S1C i). Long lasting HWs are found in the North-western South American coast and the Galápagos Islands (Fig. S1C ii). Regarding HWs’ frequency there is an overall consistent pattern of more than 20 days in a year along the Southern Hemisphere (Fig. S1C iii). Storms on land show higher mean intensity values in higher latitudes (Fig. S1D i); while their duration and frequency have been higher from mid to lower latitudes (Fig. S1D ii and iii). Antarctica also has high frequency of storms. Intense precipitations occur in South America and the Pacific Islands (Fig. S1E i). In contrast, precipitations tend to last more and be more frequent in Central Africa, the North of Australia, and the central part of Brazil (Fig. S1E ii and iii).

**Fig. S2. Trends for the different extreme events and metrics:** MHW intensity has positive and significant trends from mid to high latitudes, and negative from mid to low latitudes (Fig. S2A i). MHW duration does not show clear spatial patterns (Fig. S2A ii), whereas a general positive trend in MHW frequency is observed across the Southern Hemisphere, except around Antarctica (Fig. S2A iii). Storms at sea have no consistent spatial patterns for intensity (Fig. S2B i); whereas an overall positive trend in duration and frequency is observed in the equatorial regions of the South Pacific Ocean, except for the Peruvian coast that shows negative trends (Fig. S2B ii and iii). We also observe a positive trend in the frequency of storms at sea over the Atlantic Ocean (Fig. S2B iii). In the Antarctic Peninsula HW trends were positive in intensity and negative in frequency, while the opposite trends are observed for the Queen Maud Land (Fig. S2C i and iii). Overall, positive trends in HW frequency are also observed over the Southern Hemisphere (Fig. S2C iii). In contrast, no clear patterns are observed on HW intensity and duration (except for the Antarctic Peninsula; Fig. S2C i and ii)). Storms on land exhibit no clear patterns in terms of intensity and duration trends (Fig. S2D i and ii). We observe positive trends in the frequency of storms on land in Brazil, North Africa, and West Antarctica (Fig. S2D iii). There are also some regions with negative trends in storm on land frequencies across Guayanas and Central and Eastern Africa. Precipitation intensity has positive trends all over the Southern Hemisphere (Fig. S2E i); while no clear trends in terms of duration are observed, except for Central Africa where a positive trend emerges (Fig. S2E ii). Regarding the frequency of extreme precipitation, there is an overall negative trend except in Guayanas, the Pacific Islands and some regions in East Antarctica (Fig. S2E iii).


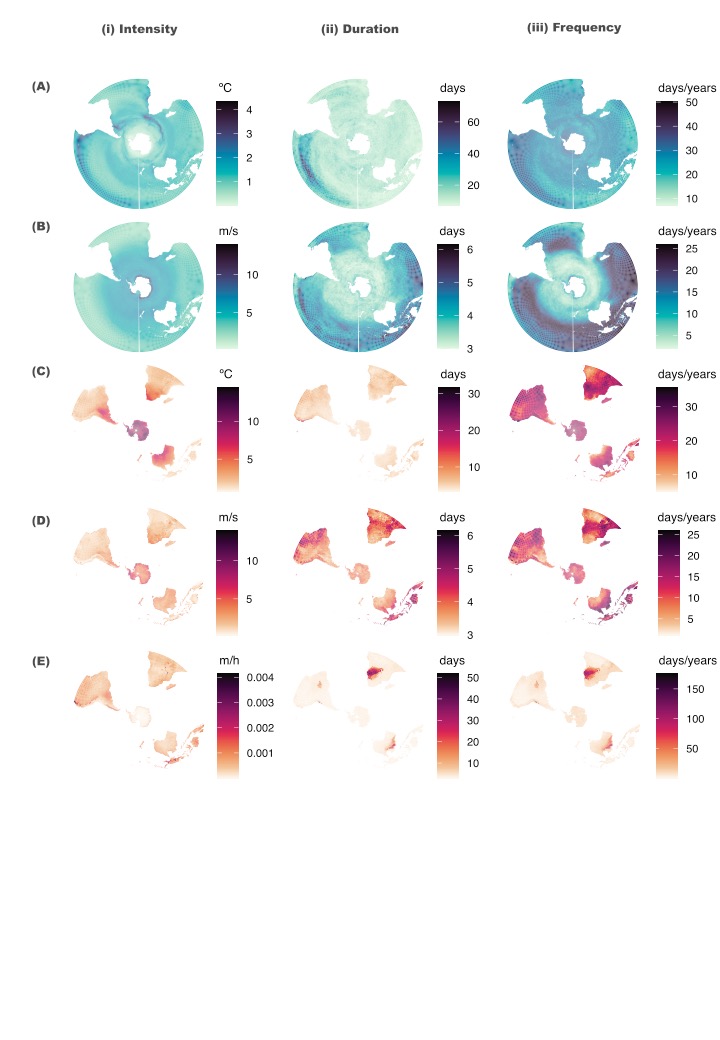


**Fig. S1.** **Mean values for the different extreme events and metrics.** Extreme events on: (**A**) Marine Heatwaves, (**B**) Extreme Winds at sea, (**C**) Land heatwaves, (**D**) Extreme Winds on land, and (**E**) Extreme Precipitation. Metrics: (**i**) Intensity, (**ii**) Duration, and (**iii**) Frequency.

**Fig. S2.** **Trends for the different extreme events and metrics.** Extreme events on: (**A**) Marine Heatwaves, (**B**) Extreme Winds at sea, (**C**) Land heatwaves, (**D**) Extreme Winds on land, and (**E**) Extreme Precipitation. Metrics: (**i**) Intensity, (**ii**) Duration, and (**iii**) Frequency.

**Table S1. How do extreme events affect penguins.** Summary of the main effects found in the literature over penguin populations.

| **Extreme event** | **Effect observed in some penguin species** | **References** |
| --- | --- | --- |
| **Marine Heatwaves** | - Changes in the diet (food web and predator-prey relationship) can lead to starvation and poor body conditions if no appropriate food is found.  **-** Impact in chick growth and fledging success.  - Carry over effect of adverse conditions in the wintering grounds.  - Unusual moulting patterns and mortality. | ^1–6^ |
| **Extreme Winds at sea** | **-** Decline in body mass during stormy periods, likely due to the increased difficulty in locating prey. Adverse effects can persist after the storm subsided.  **-** Impact in chick growth and fledging success. The effect can depend on the breeding stage. | ^7–11^ |
| **Heatwaves** | **-** Heat related mortality (moulting penguins and chicks are more vulnerable, yet also reported deaths for adults).  - Heat avoidance behaviour letting the chicks unattended. Massive incubation failure.  **-** Impact in chick growth and fledging success. | ^12–15^ |
| **Extreme Winds on land** | - Cold related mortality (moulting penguins and chicks).  **-** Impact in chick growth and fledging success.  - Nest destruction. | ^16–19^ |
| **Extreme precipitation** | - Nest flood or collapse. Chicks’ mortality.  - Cold related mortality (moulting penguins and chicks).  **-** Impact in chick growth and fledging success. | ^13,19–25^ |

**Supplementary Material References**

1. Rebstock, G. & Boersma, P. Oceanographic conditions in wintering grounds affect arrival date and body condition in breeding female Magellanic penguins. *Mar. Ecol. Prog. Ser.* **601**, 253–267 (2018).

2. Woehler, E. & Hobday, A. Impacts of marine heatwaves may be mediated by seabird life history strategies. *Mar. Ecol. Prog. Ser.* **HEAT**, (2023).

3. Vargas, F. H., Harrison, S., Rea, S. & Macdonald, D. W. Biological effects of El Niño on the Galápagos penguin. *Biol. Conserv.* **127**, 107–114 (2006).

4. Cannell, B. *et al.* Marine heatwaves affect breeding, diet and population size but not body condition of a range-edge little penguin colony. *Mar. Ecol. Prog. Ser.* **HEAT**, (2023).

5. Morgenthaler, A. *et al.* Unusual number of Southern Rockhopper Penguins, Eudyptes chrysocome, molting and dying along the Southern Patagonian coast of Argentina: pre-molting dispersion event related to adverse oceanographic conditions? *Polar Biol.* **41**, 1041–1047 (2018).

6. Piatt, J. *et al.* Mechanisms by which marine heatwaves affect seabirds. *Mar. Ecol. Prog. Ser.* **737**, 1–8 (2024).

7. Saraux, C., Chiaradia, A., Salton, M., Dann, P. & Viblanc, V. A. Negative effects of wind speed on individual foraging performance and breeding success in little penguins. *Ecol. Monogr.* **86**, 61–77 (2016).

8. Van Eeden, R., Reid, T., Ryan, P. & Pichegru, L. Fine-scale foraging cues for African penguins in a highly variable marine environment. *Mar. Ecol. Prog. Ser.* **543**, 257–271 (2016).

9. Barreau, E., Kato, A., Chiaradia, A. & Ropert-Coudert, Y. The consequences of chaos: Foraging activity of a marine predator remains impacted several days after the end of a storm. *PLOS ONE* **16**, e0254269 (2021).

10. Berlincourt, M. & Arnould, J. P. Y. Influence of environmental conditions on foraging behaviour and its consequences on reproductive performance in little penguins. *Mar. Biol.* **162**, 1485–1501 (2015).

11. Dehnhard, N. *et al.* Survival of rockhopper penguins in times of global climate change. *Aquat. Conserv. Mar. Freshw. Ecosyst.* **23**, 777–789 (2013).

12. Holt, K. A. & Boersma, P. D. Unprecedented heat mortality of Magellanic Penguins. *Ornithol. Appl.* **124**, duab052 (2022).

13. Ganendran, L. B., Sidhu, L. A., Catchpole, E. A., Chambers, L. E. & Dann, P. Effects of ambient air temperature, humidity and rainfall on annual survival of adult little penguins Eudyptula minor in southeastern Australia. *Int. J. Biometeorol.* **60**, 1237–1245 (2016).

14. Stevenson, C. & Woehler, E. J. Population Decreases in Little Penguins Eudyptula Minor in Southeastern Tasmania, Australia, Over the Past 45 Years. (2007).

15. Traisnel, G. & Pichegru, L. Possible drivers of nest usurpation in African Penguins Spheniscus demersus. *Mar. Ornithol.* **46**, 85–88 (2018).

16. Fretwell, P. T., Boutet, A. & Ratcliffe, N. Record low 2022 Antarctic sea ice led to catastrophic breeding failure of emperor penguins. *Commun. Earth Environ.* **4**, 273 (2023).

17. Fretwell, P. T. & Trathan, P. N. Emperors on thin ice: three years of breeding failure at Halley Bay. *Antarct. Sci.* **31**, 133–138 (2019).

18. Schmidt, A. E. & Ballard, G. Significant chick loss after early fast ice breakup at a high-latitude emperor penguin colony. *Antarct. Sci.* **32**, 180–185 (2020).

19. Boersma, P. D. & Rebstock, G. A. Climate Change Increases Reproductive Failure in Magellanic Penguins. *PLoS ONE* **9**, e85602 (2014).

20. Wolfaardt, A. C., Crofts, S. & Baylis, A. M. M. Efects of a storm in colonies of seabirds breeding at the falkland islands. (2012).

21. Demongin, L., Poisbleau, M., Strange, I. J. & Quillfeldt, P. Effects of severe rains on the mortality of Southern Rockhopper Penguin (Eudyptes chrysocome) chicks and its impact on breeding success. *Ornitol. Neotropical* **21**, 439–443 (2010).

22. Boersma, P. D., Rebstock, G. A., Frere, E. & Moore, S. E. Following the fish: penguins and productivity in the South Atlantic. *Ecol. Monogr.* **79**, 59–76 (2009).

23. Chilvers, B. L. & Hiscock, J. A. Significant decline of endangered Antipodes Island penguins: Is extreme weather an additional impact? *Aquat. Conserv. Mar. Freshw. Ecosyst.* **29**, 546–553 (2019).

24. Bried, J. & Jouventin, P. The King Penguin Aptenodytes patagonicus, a non‐nesting bird which selects its breeding habitat. *Ibis* **143**, 670–673 (2001).

25. Chapman, E., Hofmann, E., Patterson, D., Ribic, C. & Fraser, W. Marine and terrestrial factors affecting Adélie ­penguin (Pygoscelis adeliae) chick growth and recruitment off the western Antarctic Peninsula. *Mar. Ecol. Prog. Ser.* **436**, 273–289 (2011).
